# Multigene panel analysis identified germline mutations of DNA repair genes in breast and ovarian cancer

**DOI:** 10.1002/mgg3.157

**Published:** 2015-05-12

**Authors:** Yosuke Hirotsu, Hiroshi Nakagomi, Ikuko Sakamoto, Kenji Amemiya, Toshio Oyama, Hitoshi Mochizuki, Masao Omata

**Affiliations:** 1 Genome Analysis Center, Yamanashi Prefectural Central Hospital1-1-1 Fujimi, Kofu, Yamanashi, 400-8506, Japan; 2 Department of Breast Surgery, Yamanashi Prefectural Central Hospital1-1-1 Fujimi, Kofu, Yamanashi, 400-8506, Japan; 3 Department of Gynecology, Yamanashi Prefectural Central Hospital1-1-1 Fujimi, Kofu, Yamanashi, 400-8506, Japan; 4 Pathology Division, Laboratory Department, Yamanashi Prefectural Central Hospital1-1-1 Fujimi, Kofu, Yamanashi, 400-8506, Japan; 5 Department of Gastroenterology, Yamanashi Prefectural Central Hospital1-1-1 Fujimi, Kofu, Yamanashi, 400-8506, Japan; 6 Graduate School of Medicine, The University of Tokyo7-3-1 Hongo, Bunkyo-ku, Tokyo, 113-8655, Japan

**Keywords:** Breast and ovarian cancer, DNA repair, gene panel, next-generation sequencing

## Abstract

Approximately 5–10% of all breast and/or ovarian cancer cases are considered as inherited. *BRCA1* and *BRCA2* tumor suppressor genes account for a high penetrance of hereditary cases, but familial cases without mutations in these genes can also occur. Despite their low penetrance, other hereditary cancer-related genes are known to be associated with breast and ovarian cancer risk. However, the extent to which these genes prevail in breast and ovarian cancer remains to be elucidated. To estimate the frequency of mutations in these predisposition genes, we analyzed the germline mutations of 25 hereditary cancer-related genes in 155 patients using targeted next-generation sequencing. These subjects included 11 BRCA1/2 mutation-positive cases and 144 negative cases. Of these, three patients (1.9%) had pathogenic mutations in *ATM*, *MRE11A*, or *MSH6*, all of which have a central role in DNA repair and the mismatch repair pathway. The *MSH6* splice-site mutation (IVS6+1G>T) was predicted to be pathogenic, as demonstrated by in vitro and immunohistochemical analyses. These results suggested deficiencies in cellular DNA repair functions result in the development of breast and ovarian cancer.

## Introduction

Breast and ovarian cancer are common cancers in women, affecting ∼60,000 and 10,000 new cases, respectively, per year in Japan. Approximately 5–10% of breast and ovarian cancers are hereditary disorders. Germline mutations in *BRCA1* and *BRCA2* (*BRCA1/2*) tumor suppressor genes predispose to the development of breast and ovarian cancer (Easton et al. 1993; Narod et al. 1995; King et al. 2003), but *BRCA1/2* mutations have been observed in only about 20% of these cases (Couch et al. 2014b). A subset of the remaining cases appears to be caused by germline mutations in other cancer susceptibility genes (Couch et al. 2014b). For instance, *TP53*, *PTEN*, *CDH1*, *STK11*, *ATM*, *PALB2*, and *CHEK2* are predisposing genes for breast cancer (Economopoulou et al. 2015). In addition, mutations in DNA mismatch repair genes such as *MLH1*, *MSH2*, *MSH6*, and *PMS2*, which are responsible for Lynch syndrome (also known as hereditary nonpolyposis colorectal cancer), are associated with not only colon and endometrial cancer but also with ovarian, biliary tract, and stomach cancer (Bonai et al. 2011; Vierkoetter et al. 2014). These observations suggested that analysis of predisposition genes associated with hereditary cancer is useful in screening mutation carriers for cancer surveillance and prevention.

With the advent of high-throughput sequencing technologies, next-generation sequencing (NGS), it is possible to simultaneously analyze multiple genes of interest at low cost (Tung et al. 2015). NGS is gradually making its way into clinical research for diagnostic testing of hereditary disorders (Domchek et al. 2013; Grant et al. 2014; LaDuca et al. 2014; Yorczyk et al. 2014). Previous reports demonstrating NGS analysis of hereditary cancer-related genes identified germline mutations in breast and ovarian cancer patients (Walsh et al. 2011; Couch et al. 2014a; Cybulski et al. 2014; Kurian et al. 2014). However, the extent to which hereditary cancer genes other than *BRCA1/2* are responsible for breast and ovarian cancer remains to be fully elucidated. In this study, we selected 25 cancer-predisposing genes, and performed targeted sequencing in 155 breast and/or ovarian cancer subjects to identify mutation carriers and assess the frequency of mutations in these predisposition genes.

## Materials and Methods

### Patients and sample preparation

Peripheral blood samples were obtained from 155 breast and/or ovarian cancer patients who attended the Yamanashi Prefectural Central Hospital (Yamanashi, Japan) between 2013 and 2014. This cohort included 76 patients with breast cancer, 69 with ovarian cancer, and 10 with both breast and ovarian cancers and the median age of cancer onset was 55 years (range, 16–87 years). According to the National Comprehensive Cancer Network (NCCN) criteria, 146 patients (94%) had genetic risks based on the family history. Lymphocytes were isolated following centrifugation of peripheral blood samples at 820*g* at 25°C for 10 min. Peripheral blood lymphocytes were stored at −80°C until required for DNA extraction. Total DNA was extracted from lymphocytes using the QIAamp DNA Blood Mini kit (Qiagen, Tokyo, Japan) or QIAamp DNA Blood Mini QIAcube Kit (Qiagen) with the QIAcube (Qiagen). The concentration of DNA was determined using the Nano Drop 2000 spectrophotometer (Thermo Fisher Scientific, Yokohama, Japan). Informed consent was obtained from all subjects, and this study was approved by the Institutional Review Board at Yamanashi Prefectural Central Hospital.

### Targeted NGS

For targeted NGS analysis, Ion AmpliSeq designer software (Life Technologies, Tokyo, Japan) was used to design primers, which consisted of 610 primer pairs in two pools covering the exons and exon–intron boundaries of 25 cancer-predisposing genes (*APC*, *ATM*, *BARD1*, *BMPR1A*, *BRIP1*, *CDH1*, *CDK4*, *CDKN2A*, *CHEK2*, *EPCAM*, *MLH1*, *MRE11A*, *MSH2*, *MSH6*, *MUTYH*, *NBN*, *PALB2*, *PMS2*, *PTEN*, *RAD50*, *RAD51C*, *RAD51D*, *SMAD4*, *STK11*, and *TP53*) (Walsh et al. 2011; Couch et al. 2014b; Economopoulou et al. 2015; Tung et al. 2015). Multiplex polymerase chain reaction (PCR) was performed using 50–100 ng genomic DNA with 17 cycles with a premixed primer pool using Ion AmpliSeq Library Kit 2.0, as previously described (Hirotsu et al. 2014). The PCR amplicons were treated with 2 *μ*L FuPa reagent to partially digest primer sequences and phosphorylate the amplicons. The amplicons were ligated to adapters with the diluted barcodes of the Ion Xpress Barcode Adapters kit (Life Technologies). Adaptor-ligated amplicon libraries were purified using Agencourt AMPure XP reagents (Beckman Coulter, Tokyo, Japan). The library concentration was determined using an Ion Library Quantitation Kit (Life Technologies), then each library was diluted to 8 pM and the same amount of libraries was pooled for one sequence reaction. Next, emulsion PCR was carried out using the Ion OneTouch System and Ion PI Template OT2 200 Kit v2 (Life Technologies) according to the manufacturer’s instructions. Template-positive Ion Sphere Particles were then enriched with Dynabeads MyOne Streptavidin C1 Beads (Life Technologies) using an Ion OneTouch ES system (Life Technologies). Purified Ion Sphere particles were loaded on an Ion PI Chip v2. Massively parallel sequencing was carried out on an Ion Proton System (Life Technologies) using the Ion PI Sequencing 200 Kit v2. Sequencing was performed using 500 flow runs that generated ∼200 bp reads.

### Data analysis

The sequence data were processed using standard Ion Torrent Suite Software running on the Torrent Server. Raw signal data were analyzed using Torrent Suite version 4.2. The pipeline included signaling processing, base calling, quality score assignment, adapter trimming, PCR duplicate removal, read alignment to human genome 19 reference (hg19), quality control of mapping quality, coverage analysis, and variant calling. Following data analysis, annotation of single nucleotide variants, insertions, deletions, and splice-site alternations were performed by the Ion Reporter Server System (Life Technologies). Splice-site alternations were analyzed 2 bp upstream or downstream of exon–intron boundaries. Sequence data were visually confirmed with the Integrative Genomics Viewer (IGV) and any sequence, alignment, or variant call error artifacts were discarded. Heterozygous *MUTYH* mutations were not considered as pathogenic in this study. Public databases used included ClinVar (Landrum et al. 2014), the 1000 Genomes Project database (Abecasis et al. 2012), the 5000 Exome project (http://evs.gs.washington.edu/EVS/), and The Human Genetic Variation Browser (HGVB) (http://www.genome.med.kyoto-u.ac.jp/SnpDB).

### Sanger sequencing

PCR was performed using genomic DNA and primer pairs flanking the deleterious variant sites. PCR products were purified using the QIAquick PCR Purification Kit (Qiagen). Sequencing was performed with BigDye Terminator v3.1 (Life Technologies) using forward or reverse primers. PCR products were purified and subsequently analyzed by the 3500 Genetic Analyzer (Applied Biosystems, Tokyo, Japan). Primer sequences are provided in Table S1.

### Reverse transcriptase-PCR

Total RNA from peripheral blood was extracted using the NucleoSpin RNA Blood kit (Takara, Shiga, Japan) and reverse transcribed to cDNA using High-Capacity cDNA Reverse Transcription Kits (Applied Biosystems). PCR was performed using Platinum PCR SuperMix High Fidelity (Life Technologies) with primer pairs listed in Table S1. The PCR products were separated on a 2% agarose gel.

### Immunohistochemical analysis

The sections were deparaffinized, and antigen retrieval was performed by heat treatment in EDTA (ethylenediaminetetraacetic acid) solution pH 8.0. MSH6 protein expression in tumors and surrounding normal tissue was evaluated on 4-*μ*m-thick, formalin-fixed, paraffin-embedded (FFPE) sections with anti-MSH6 monoclonal antibodies (clone 44; 1:400; BD Biosciences, Tokyo, Japan) using the Ventana BenchMark XT staining system (Roche, Tokyo, Japan). Normal tissue adjacent to tumor tissue served as a positive control. A pathologist determined the tumors to be positive when nuclear staining in tumor tissue was present or negative when the nuclear stain was absent.

### MSI analysis

The tissue was stained with hematoxylin–eosin and then microdissected using an ArcturusXT laser-capture microdissection system (Life Technologies). DNA was extracted from FFPE tumor tissue using the QIAamp DNA FFPE Tissue Kit (Qiagen). Peripheral blood DNA was used as a control. For microsatellite instability (MSI) analysis, five microsatellite markers (BAT25, BAT26, D5S346, D2S123, and D17S250) were used to classify the tumor as MSI-high (MSI-H, the presence of at least two markers showing novel alleles compared with normal tissue), MSI-low (defined as one marker with a novel allele), or microsatellite stable (MSS, no marker with novel alleles) (Berg et al. 2000). Fluorescently labeled PCR products were separated by capillary electrophoresis using a 3500 Genetic Analyzer (Applied Biosystems) and the product size was analyzed by GeneMapper Software 5 (Applied Biosystems). Primer sequences are provided in Table S1.

## Results

### Sequencing multiple genes related to hereditary cancer

We selected 25 hereditary cancer-related genes not including *BRCA1/2* and designed a customized gene panel, which covered 97.6% of target regions in these genes (Table S2). Massively parallel sequencing determined the sequence of coding regions and intron–exon boundaries of these genes in lymphocyte DNA from 155 breast and/or ovarian cancer patients. These subjects included 11 *BRCA1/2* mutation-positive cases and 144 negative cases (Hirotsu et al. 2014). Target sequencing was conducted using an Ion Proton sequencer. Each library yielded an average of 10.5 Gb of total number of bases aligned to reference sequence (Table S3). The mean sequencing depth was 2805×, and the average uniformity of coverage was 90.2% (Table S4).

As a result, we identified 10 inactivating mutations in *ATM*, *MRE11A*, *MSH6*, and *MUTYH* genes in nine patients who did not carry *BRCA1/2* mutations (Table1). These mutations are expected to cause protein truncations through frameshift insertions or deletions (*n* = 3), nonsense mutations (*n* = 1), or splice-site alterations (*n* = 6) (Table1). Among these variants, *MSH6* frameshift mutation (p.K1358fs) is predicted to be nonpathogenic because the mutation causes the deletion of two amino acids at the C-terminal region (*MSH6* full length: 1360 amino acids) and may not affect protein function (Fig. S1). Furthermore, a splice site mutation in MSH6 (IVS9+2_+5delGTAAC>G) was also supposed to be nonpathogenic, because the consensus dinucleotide GT was retained despite the deletion of four nucleotides (TAAC) (Fig. S2). The frequencies of the MSH6 p.K1358fs and MSH6 IVS9+2_+5delGTAAC>G variants in a Japanese population were 1.8% and 1.3%, respectively, according to HGVB (Table1), supporting the theory that these variants are relatively common and nonpathogenic. *MUTYH* is considered as a predisposing gene for MYH-associated polyposis coli (MAP), an autosomal recessive disease (Al-Tassan et al. 2002). In this study, four patients had *MUTYH* monoallelic mutations in the splice site (Table1), but biallelic mutations were not identified.

**Table 1:** Mutations in hereditary cancer-related genes in 155 patients with breast and/or ovarian cancer

Patient no.	Personal history (age at diagnosis)	Gene	Chr	Position	Designation	Coding	Variant Allele Fraction	1000 genome MAF	HGVB	ClinVar
1	Breast (65)	*MRE11A*	11	94212003	p.G148X	c.442G>T	0.45	ND	ND	ND
2	Breast (44)	*ATM*	11	108203577	p.I2629fs	c.7878_7882delTTATA	0.48	ND	ND	ND
3	Breast (59)	*MSH6*	2	48033981	p.K1358fs	c.4065_4066insTTGA	0.49	0.8%	1.8%	ND
4	Ovary (cc) (57) Breast	*MSH6*	2	48033981	p.K1358fs	c.4065_4066insTTGA	0.45	0.8%	1.8%	ND
5	Breast (41)	*MSH6*	2	48033791	IVS9+2_+5delGTAAC>G	splice site	0.46	ND	1.3%	ND
	Ovary (muc) (56)									
6	Ovary (endo) (47)	*MSH6*	2	48032167	IVS6+1G>T	splice site	0.43	ND	ND	ND
		*MUTYH*	1	45797760	IVS10-2A>G	splice site	0.37	0.2%	2.6%	Likely pathogenic
	Uterine corpus (47)									
7	Breast (46)	*MUTYH*	1	45797760	IVS10-2A>G	splice site	0.44	0.2%	2.6%	Likely pathogenic
8	Ovary (59)	*MUTYH*	1	45797760	IVS10-2A>G	splice site	0.41	0.2%	2.6%	Likely pathogenic
9	Breast (43)	*MUTYH*	1	45797760	IVS10-2A>G	splice site	0.39	0.2%	2.6%	Likely pathogenic

Chr, chromosome; MAF, minor allele frequency; cc, clear cell; muc, mucinous; endo, endometrioid; ND, not documented; HGVB, human genetic variation browser.

**Figure 1:**
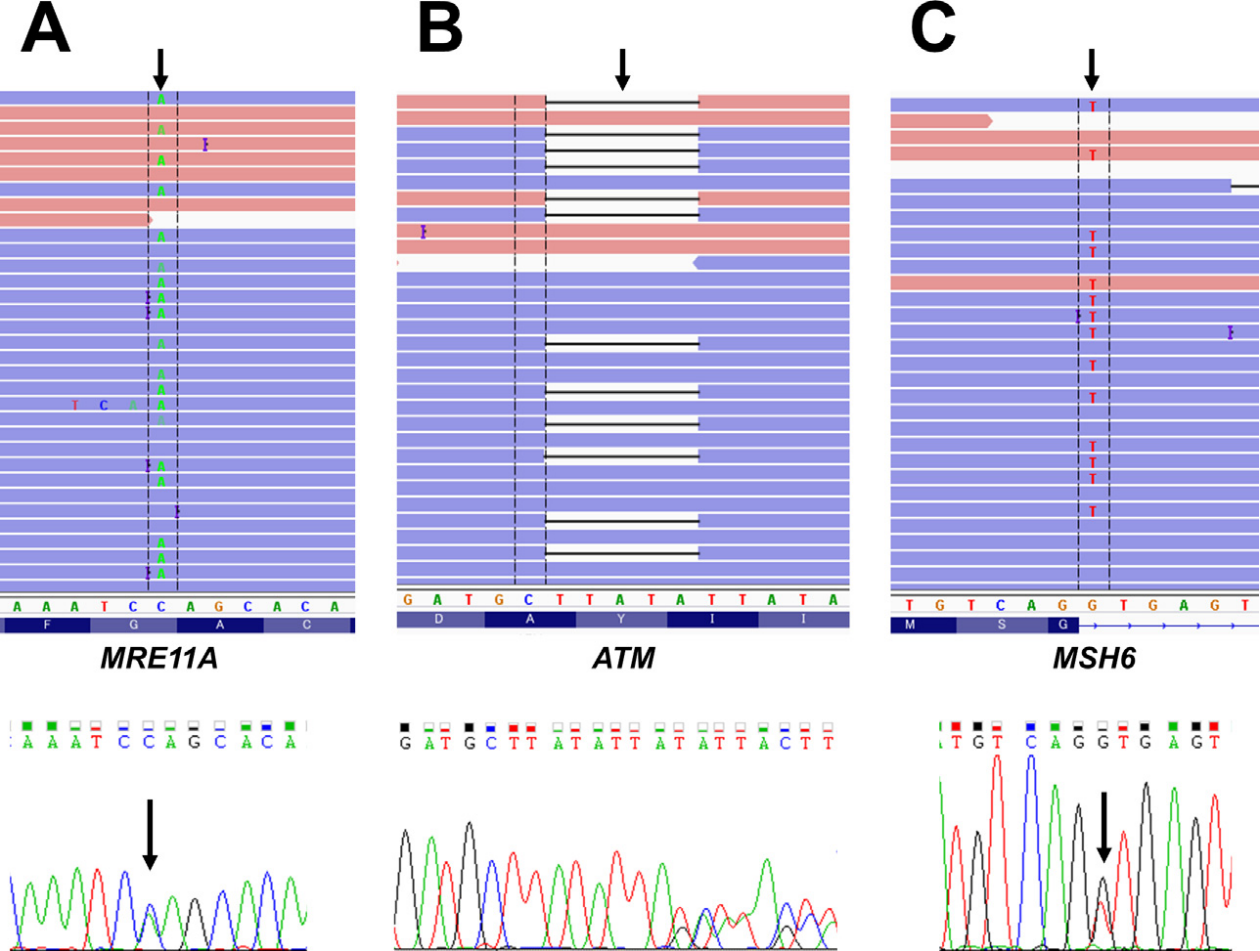
Germline deleterious mutations identified in patients with breast and/or ovarian cancer. Representative image of read alignments visualized with IGV (upper image). Sequencing chromatograms show the mutations and frameshift insertions/deletions in peripheral blood DNA from each patient (lower image). Mutations were identified in *MRE11A* p.G148X (A), *ATM* p.I2629fs (B), and *MSH6* IVS6+1G>T (C). Arrows indicate the position of the mutations in the patient genome.

Therefore, we concluded that deleterious mutations in *BRCA1/2* (7.1%; 11 out of 155) genes and in non-*BRCA1/2*-predisposing genes (1.9%; three out of 155) were present in our subjects (Fig.1 and Table S5). All deleterious mutations in *MRE11A*, *ATM*, and *MSH6* genes were confirmed by Sanger sequencing (Fig.1). Taken together, the data suggested a subpopulation of breast and ovarian cancer has a germline mutation related to DNA repair genes.

### Deleterious mutation in predisposing genes

**Figure 2:**
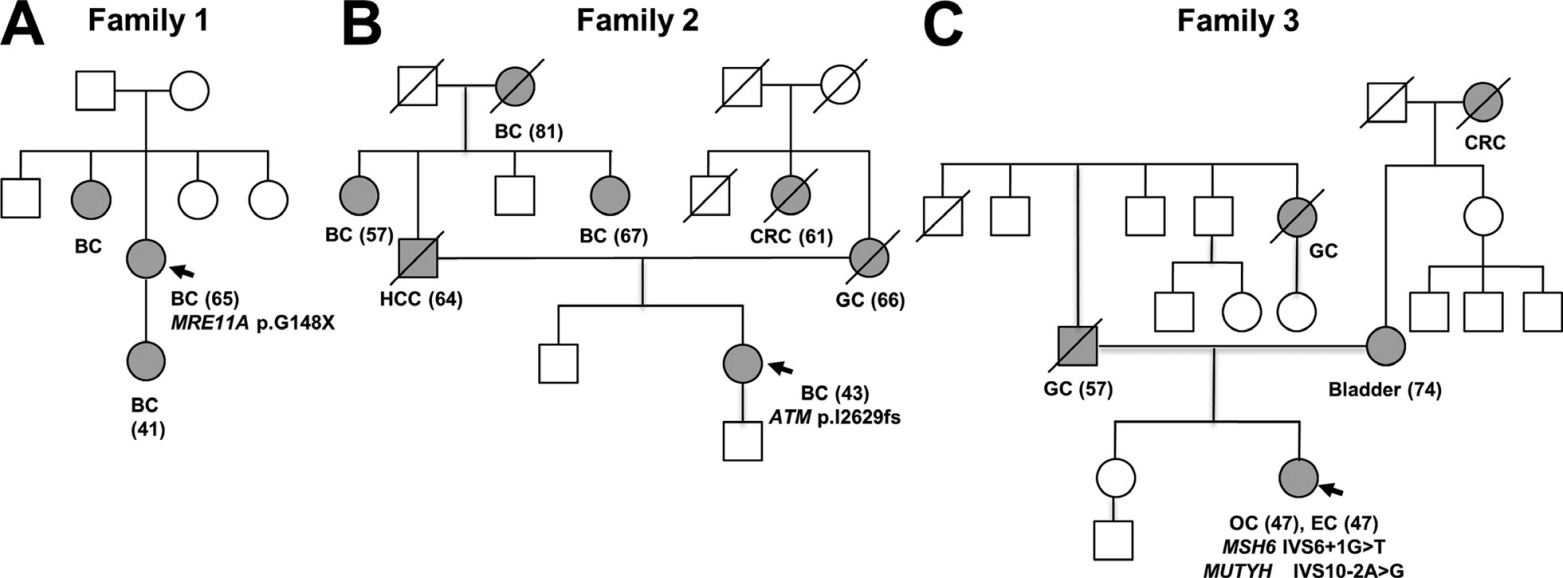
Pedigrees of three families with deleterious mutations. Individuals with any cancer are shown as filled circles. Arrows show a proband with deleterious mutations of (A) *MRE11A* in family 1, (B) *ATM* in family 2 and (C) *MSH6* in family 3. The age at diagnosis plus the identified mutation is shown under the relevant individuals. BC, breast cancer; OC, ovarian cancer; CRC, colorectal cancer; GC, gastric cancer; HCC, hepatocellular cancer; Bladder, bladder cancer; EC, endometrial cancer.

We identified *MRE11A* nonsense (p.G148X) or *ATM* frameshift (p.I2629fs) mutations in probands who developed breast cancer (Table1). The mutations in *ATM* and *MRE11A* were identified in breast cancer patients (Broeks et al. 2000; Bartkova et al. 2008). Family 1 included a heterozygous *MRE11A* nonsense mutation (p.G148X) that was present in probands and two relatives with breast cancer (Fig.2A). Family 2 included two aunts and one grandmother who developed breast cancer. A heterozygous *ATM* frameshift mutation (p.Ile2629fs) was identified in a proband who developed breast cancer at the age of 43 years (Fig.2B).

In addition, a deleterious splice-site mutation in *MSH6* was detected in ovarian and endometrial cancer patients, and ovarian cancer was diagnosed with mucinous carcinoma (Table1). Consistent with the evidence that *MSH6* is associated with Lynch syndrome, family 3 included one grandfather who developed colorectal cancer (Fig.2C). Germline mutations in other mismatch repair genes such as *MLH1*, *MSH2*, or *PMS2* were not identified in this study.

### Mutation in *MSH6* genes (IVS6+1G>T) were pathogenic

**Figure 3:**
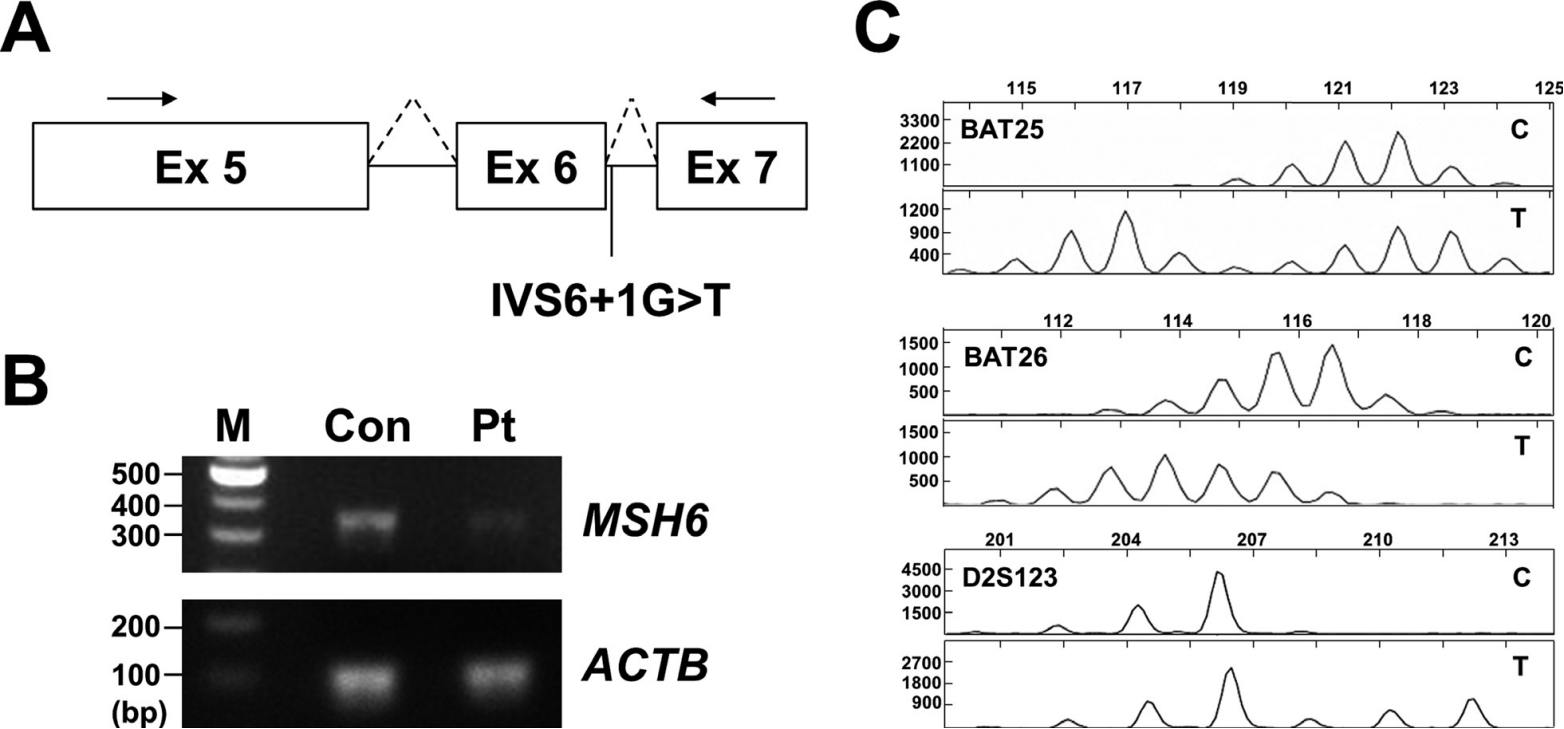
MSH6 splice-site mutation is pathogenic. (A) Schematic of *MSH6* transcript. The arrows indicate the primers used in this analysis, located in exon (Ex) 5 and exon 7. (B) Reverse transcriptase polymerase chain reaction analysis of RNA from peripheral blood of a control (Con) and patient (Pt) with ovarian and endometrial cancer harboring the *MSH6* IVS6+1G>T splice-site mutation. Gel electrophoresis showing PCR fragments (∼356 bp in length). DNA marker (M) was loaded in the left lane. *ACTB* was used as an internal control. (C) Representative images of MSI profiles. Ovarian tumor showing microsatellite instability for BAT25, BAT26, and D2S123. C, control; T, tumor tissue; IVS, intervening sequence; MSI, microsatellite instability.

**Figure 4:**
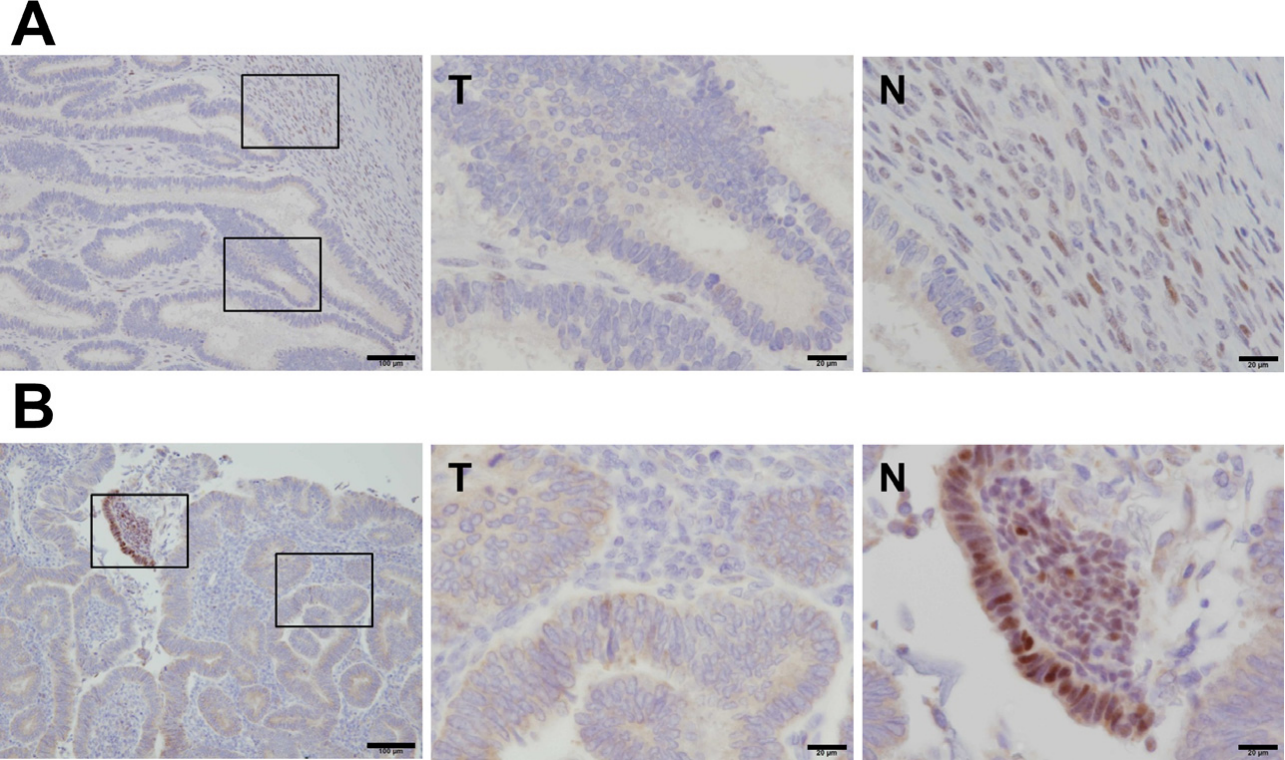
MSH6 proteins are downregulated in ovarian and endometrial cancer. (A and B) Immunohistochemical staining of tumor (T) and surrounding normal (N) tissue with an MSH6 antibody in ovarian (A) and endometrial cancer (B). MSH6 protein accumulated in the nuclei of normal tissue (left image), but not in tumor tissue (middle image). Right-hand images show a high-power magnification of left-hand images. Scale bars, 100 *μ*m for left-hand image, and 20 *μ*m for middle and right-hand image. N, normal tissue; T, tumor tissue.

The splice-site mutation in *MSH6* (IVS6+1G>T) disrupts the canonical GT dinucleotide at the 5′ splice site and has not been reported previously to our knowledge. In addition, loss of MSH6 promotes tumorigenesis in colorectal cancer is well known, but not in ovarian cancer. To examine whether this G to T substitution influences transcriptional processing, we synthesized *MSH6* complementary DNA (cDNA) from peripheral blood RNA and performed PCR with primers flanking exon 6 (Fig.3A). The PCR product sizes were ∼356 bp in length, which represented the normal transcript of exon 5–7 expressed in both controls and patients (Fig.3B). However, *MSH6* mRNA expression levels were apparently reduced in patients compared with controls (Fig.3B). These results suggested the splice-site mutation introduced premature termination codons and subsequently mRNA was degraded by nonsense-mediated decay. We next performed MSI analysis and immunohistochemistry staining for MSH6. This revealed that the tumors had MSI-high and lacked MSH6 protein expression (Figs.3C, 4). These results showed the *MSH6* IVS6+1G>T variant disrupted mismatch-repair function and promoted tumorigenesis.

## Discussion

In this study, we performed NGS using a multigene panel to estimate the frequency of germline mutation carriers with breast and/or ovarian cancer. Of 155 patients, three had pathogenic mutations in *ATM*, *MRE11A*, or *MSH6*. These results suggested individuals carrying germline mutations in DNA repair genes were at risk of breast or ovarian cancer. Thus, instead of single-gene testing, multigene panel-based genetic testing is an alternative tool for screening hereditary cancer.

DNA repair genes maintain genomic stability in response to DNA damage to retain cellular homeostasis. The MER11 complex (MRE11–RAD50–NBS1: MRN) binds double-strand break ends and activates ataxia-telangiectasia-mutated (ATM) protein (Lee and Paull 2005). Homozygous or compound heterozygous mutations in *ATM* or *MRE11A* cause ataxia-telangiectasia (MIM: 208900) and ataxia-telangiectasia-like disorder-1 (ATLD1) (MIM: 604391) (Savitsky et al. 1995; Stewart et al. 1999). *ATM* is a predisposing gene for breast and pancreatic cancer (Broeks et al. 2000; Roberts et al. 2012).

Of note, we identified heterozygous germline mutations in these two genes in breast cancer but not ovarian cancer patients, which implies that defects in the MRN–ATM signaling pathway result in the development of breast cancer. A previous study demonstrated *ATM* heterozygous mutation carriers have a two-fold increased risk of breast cancer compared with the general population, and in particular, women under the age of 50 years have a five-fold increased risk (Thompson et al. 2005). Consistent with this, our analysis showed a proband carrying an *ATM* germline mutation developed breast cancer at the age of 43 years (Fig.2B). Moreover, this proband had three relatives with second-degree relatives who developed breast cancer. Despite limited evidence, mutations in *MRE11A* are associated with breast cancer (Bartkova et al. 2008). Our data showed a proband carrying a *MRE11A* mutation had two relatives who developed breast cancer, reinforcing the evidence that *MRE11A* is a predisposing gene for breast cancer.

Germline mutations in mismatch repair genes, including *MLH1*, *MSH2*, *MSH6*, and *PMS2*, predispose to Lynch syndrome. Defects in *MLH1* (50%) and *MSH2* (40%) account for the majority of Lynch syndrome cases, but mutations in *MSH6* (∼7–10%) and *PMS2* (<5%) are responsible for the minority of cases (Hegde et al. 2013). This study identified one type of *MSH6* germline mutation in ovarian cancer cases. Unlike Lynch syndrome, mutations in *MSH2* and *MLH1* genes were not observed in our study. Walsh et al. (2011) also reported mutations in *MSH6* but not in *MLH1* or *MSH2* in ovarian cancer patients. Collectively, it is possible that *MSH6* among the mismatch repair genes was strongly associated with ovarian cancer.

In conclusion, despite the low frequency of the deleterious mutations in non-*BRCA1/2* predisposing genes, multigene panel genetic testing is an alternative tool for screening familial cancer patients in the clinic. Discovery of deleterious mutations by NGS has potential clinical utility both for individuals with cancer and their relatives. For instance, *ATM* and *MRE11A* deficiency is sensitive to poly (ADP-ribose) polymerase-1 (PARP-1) inhibition (Williamson et al. 2010; Vilar et al. 2011; Ledermann et al. 2014). In addition, clinical intervention such as salpingo-oophorectomy, which is undertaken in many women with *BRCA* mutations, may decrease cancer risk in probands and in their relatives with mutations (Munsell et al. 2006; Rebbeck et al. 2009). Therefore, breast and/or ovarian cancer patients without *BRCA1/2* mutations should be screened using panel-based analyses for cancer prevention during routine medical checkups.

## Supporting Information

**Table S1.** Primer sequencings. **Table S2.** Gene list in multi-gene panel. **Table S3.** Run summary. **Table S4.** Coverage data. **Table S5.** Deleterious mutation identified in this analysis.

**Figure S1.** *MSH6* K1358fs (c.4065_4066insTTGA) mutation is suspected to be a nondeleterious mutation. Representative image of read alignments visualized with IGV; the arrow (purple bar in image) indicates the insertion site (upper image). Sequencing chromatograms show the frameshift insertions in peripheral blood DNA (lower image). Nucleotide sequence and amino acid alignment of wild-type and mutant *MSH6* (amino acid position: 1353–1360). Underlining indicates the insertion site. **Figure S2.** MSH6 splice-site mutation (IVS9+2_+5delGTAAC>G) is suspected to be nondeleterious. Schematic representation of *MSH6* wild-type and splice-site variant. The consensus dinucleotide GT is underlined. Representative image of read alignments visualized with IGV; the arrow indicates the deletion site (upper image). Sequencing chromatograms showing deletions in peripheral blood DNA from the patient (lower image).
