# Supplementary material for: Multigene panel analysis identified germline mutations of DNA repair genes in breast and ovarian cancer
Source: Mol Genet Genomic Med. 2015 May 12;3(5):459–66. doi: 10.1002/mgg3.157 (PMC4585454; doi:10.1002/mgg3.157)
Supplement: Supplementary file 2 [file mgg30003-0459-sd2.pdf]

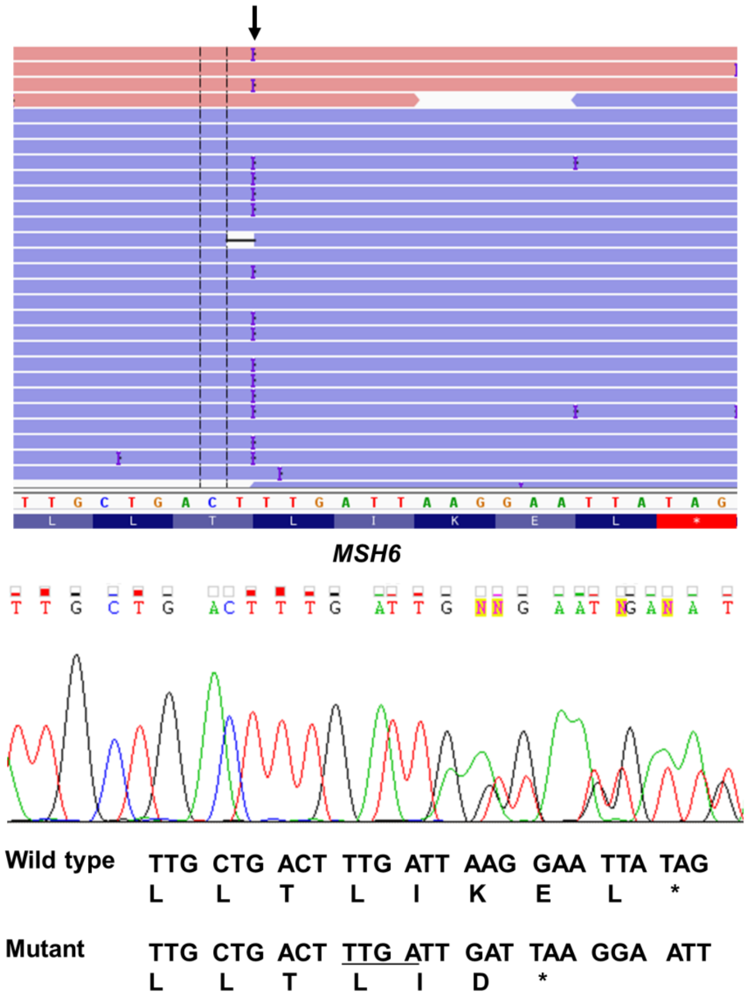

Supp. Figure S1. *MSH6* K1358fs (c.4065_4066insTTGA) mutation is suspected to be a non-deleterious mutation. Representative image of read alignments visualized with IGV; the arrow (purple bar in image) indicates the insertion site (upper image). Sequencing chromatograms show the frameshift insertions in peripheral blood DNA (lower image). Nucleotide sequence and amino acid alignment of wild-type and mutant *MSH6* (amino acid position: 1353–1360). Underlining indicates the insertion site.

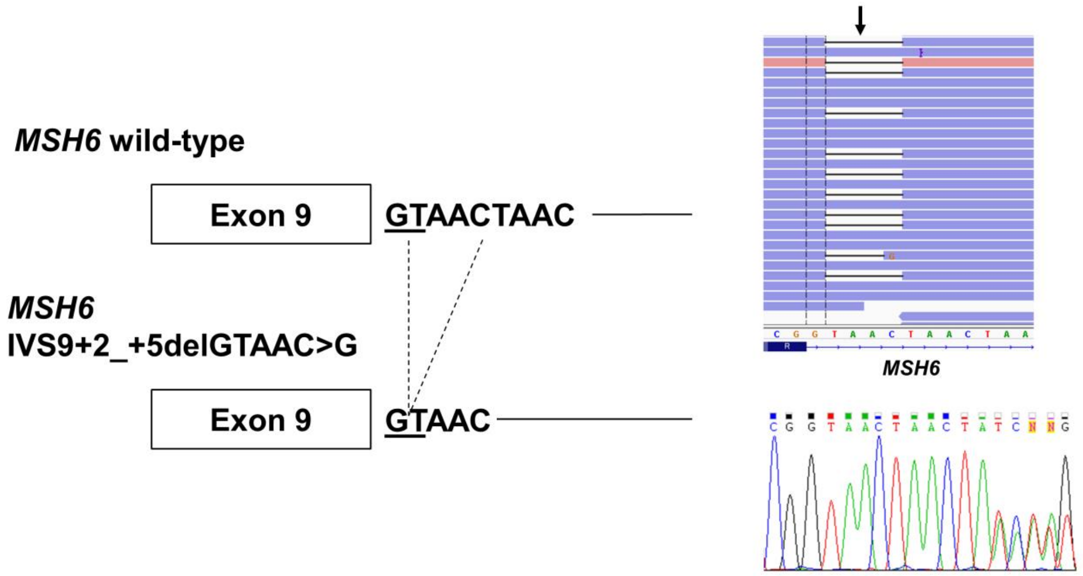

Supp. Figure S2. MSH6 splice-site mutation (IVS9+2_+5delGTAAC>G) is suspected to be non-deleterious. Schematic representation of *MSH6* wild-type and splice-site variant. The consensus dinucleotide GT is underlined. Representative image of read alignments visualized with IGV; the arrow indicates the deletion site (upper image). Sequencing chromatograms showing deletions in peripheral blood DNA from the patient (lower image).

Supp. Tables S1-S5 are available as a separate Excel file under the Supporting Information for this article.
